# Technical Note: Contrast free angiography of the pulmonary vasculature in live mice using a laboratory x-ray source

**DOI:** 10.1118/1.4964794

**Published:** 2016-10-18

**Authors:** Chaminda R. Samarage, Richard Carnibella, Melissa Preissner, Heather D. Jones, James T. Pearson, Andreas Fouras, Stephen Dubsky

**Affiliations:** 4Dx Limited, Melbourne 3000, Australia; 4Dx Limited, Melbourne 3000, Australia and Department of Mechanical and Aerospace Engineering, Monash University, Melbourne 3800, Australia; Department of Mechanical and Aerospace Engineering, Monash University, Melbourne 3800, Australia; Department of Medicine and the Women’s Guild Lung Institute, Cedars-Sinai Medical Center, Los Angeles, California 90048; Department of Physiology, Monash University, Melbourne 3800, Australia; Monash Biomedical Imaging Facility Monash University, Melbourne 3800, Australia; and Australian Synchrotron, Melbourne 3168, Australia; 4Dx Limited, Melbourne 3000, Australia and Department of Mechanical and Aerospace Engineering, Monash University, Melbourne 3800, Australia

**Keywords:** pulmonary vasculature, x-ray, angiography, contrast free, lab-source

## Abstract

**Purpose:**

*In vivo* imaging of the pulmonary vasculature in small animals is difficult yet highly desirable in order to allow study of the effects of a host of dynamic biological processes such as hypoxic pulmonary vasoconstriction. Here the authors present an approach for the quantification of changes in the vasculature.

**Methods:**

A contrast free angiography technique is validated *in silico* through the use of computer-generated images and *in vivo* through microcomputed tomography (μCT) of live mice conducted using a laboratory-based x-ray source. Subsequent image processing on μCT data allowed for the quantification of the caliber of pulmonary vasculature without the need for external contrast agents. These measures were validated by comparing with quantitative contrast microangiography in the same mice.

**Results:**

Quantification of arterial diameters from the method proposed in this study is validated against laboratory-based x-ray contrast microangiography. The authors find that there is a high degree of correlation (*R* = 0.91) between measures from microangiography and their contrast free method.

**Conclusions:**

A technique for quantification of murine pulmonary vasculature without the need for contrast is presented. As such, this technique could be applied for longitudinal studies of animals to study changes to vasculature without the risk of premature death in sensitive mouse models of disease. This approach may also be of value in the clinical setting.

## 1. INTRODUCTION

Diseases of the lungs often involve derangements of complex physiological processes that cannot be effectively studied *in vitro* or in postmortem samples. In particular, *in vivo* imaging of the pulmonary vasculature and of dynamic processes such as hypoxic pulmonary vasoconstriction has proven to be challenging in small animals. Although recent advances such as the murine thoracic-window model and oxygen-saturation mapping techniques have added greatly to the ability to image pulmonary vascular responses *in vivo*, these approaches are technically challenging and only provide information on a small area of the lung for each animal studied.^1,2^ Other techniques that have been used to quantify the distribution of pulmonary blood flow in small animals include injection of fluorescent microspheres and postmortem histological quantification,^3^ microcomputed tomography (μCT) imaging of the lungs with contrast agents either *in vivo*^4^ or after radiopaque silicone polymer injection followed by *ex vivo* lung imaging,^5^ and microangiography in mice using synchrotron radiation.^6^ However, most of these methods preclude repeated imaging due to the terminal nature of the imaging procedures and therefore prevent analysis of changes in the pulmonary distribution of blood flow over time or in correlation with changes in pulmonary lung function.

Vessel segmentation is a perennial problem in imaging research that has resulted in numerous studies.^7,8^ However, much of this work has focused on human data.^9,10^ Here, we present tools using a Hessian-based enhancement filter^11^ to obtain quantitative vessel caliber measures from μCT images without contrast. Our aim was to assess the correlation between our contrast-free caliber measures and automated caliber measures from 2D contrast microangiography. We began by calibrating our measures using synthetic images, and then applied our technique to μCT images of live mice obtained using a laboratory x-ray source. Our method shows a high degree of correlation with quantitative 2D contrast microangiography in the same mice. Thus, for the first time, this method allows volumetric measurements of the entire pulmonary vasculature in mice in 3D, without the use of contrast agents or euthanasia.

## 2. MATERIALS AND METHODS

### 2.A. Experimental procedure

Eight-week old BALB/c female mice (*n* = 5) were obtained from MARP (Monash University Research Platform, Monash University, VIC, Australia). All experiments were approved by the local Animal Ethics Committee of Monash University (Melbourne, VIC, Australia) and conducted in accordance with the guidelines set out in the Australian code of practice for the care and use of animals for scientific purposes. Mice were anesthetized with intraperitoneal injections of a mix of ketamine (Parnell Australia Pty Ltd., Alexandria NSW, Australia) and xylazine (Xylazil-20, Troy Laboratories Pty Ltd., Smithfield NSW, Australia) with doses of 150 mg/kg and 10 mg/kg, respectively. Mice were orotracheally intubated and allowed to continue breathing spontaneously while a customized 24-gauge BD Angiocath catheter (Becton-Dickinson, NJ, USA) was inserted into the jugular vein and advanced into the superior vena cava for administering contrast agents. The mouse was securely restrained in a custom-built acrylic chassis^12^ in a supine position during the surgical procedure. Mice were then ventilated using pressure control ventilation on a mouse ventilator (AccuVent200 Small Animal Ventilator, Notting Hill Devices, Melbourne, VIC, Australia) with an inspiratory pressure of 20 cm H_2_0, zero positive end-expiratory pressure, and inspiratory and expiratory times of 300 ms each (a respiratory rate of 100 breaths/min). Tidal volumes with these settings were approximately 400 *μ*l for a 20-g mouse or 20*μ*l/g (20 ml/kg). Mice were given a subcutaneous bolus of 100 *μ*l saline twice and ventilated for 10 min prior to imaging to allow equilibration and lung recruitment. Mice were kept warm using pocket warmers wrapped around the lower abdomen and legs.

### 2.B. Imaging protocol

**FIG. 1.**
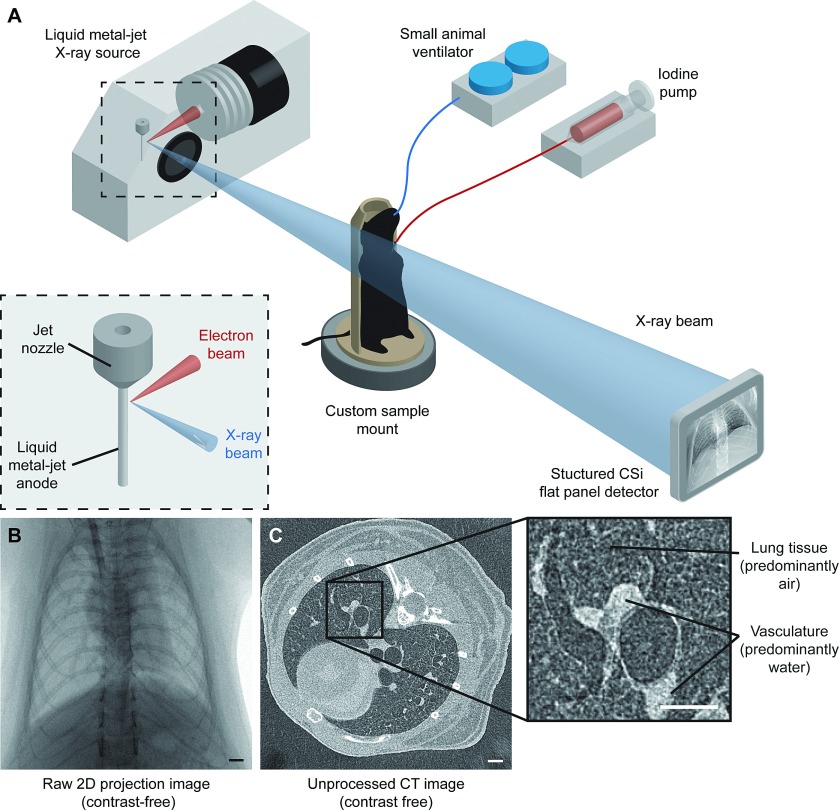
X-ray imaging setup using a laboratory source. (A) BALB/c mice, intubated and mechanically ventilated, were placed in a custom sample mount on a rotating stage in between the source and detector. For 2D microangiography, an iodinated contrast agent was injected using a microsyringe pump during a breath-hold at peak inspiration, and images were captured from 3 views. For microcomputed tomography, the mouse was rotated while images were captured in synchronization with mechanical ventilation. (B) Raw 2D projection image of a frontal view using the laboratory x-ray imaging system described in (A). (C) Single slice from an unprocessed CT volume reconstructed using a cone-beam reconstruction technique. The technique presented in this study utilizes the contrast between air-filled lung tissue (see inset) and adjacent vasculature to quantify the vessel caliber. Scale bars represent 1 mm.

Imaging was conducted in the Laboratory for Dynamic Imaging at Monash University (Melbourne, VIC, Australia). The x-ray imaging setup [see Fig. 1(A)] consists of a high brightness x-ray source (Excillum AB, Kista, Sweden) that uses an x-ray beam generated from a liquid-metal-jet microfocus (15 μm spot size) x-ray source.^13,14^ This x-ray source (70 kV, 265 W) is polychromatic and unfiltered resulting in a characteristic peak at 25 keV. A high speed CMOS flat-panel detector (PaxScan, Varian Medical Systems, Palo Alto, CA, USA) with an isotropic pixel size of 0.194 mm was used to capture images at a frame rate of 30 Hz and an exposure time of 15 ms. The mouse was positioned in the acrylic chassis in front of the x-ray beam in the upright position. A high precision rotary stage (Zaber Technologies, Vancouver, Canada) was used to rotate the mice 360° under mechanical ventilation for the CT scan. The imaging was synchronized with ventilation and gated to obtain 800 projection images of the lungs at peak inspiration for CT reconstruction. The radiation dose delivered (equating to 800 projections with 15 ms exposure times and an air kerma rate of 5.01 mGy s^−1^) was measured to be 60.12 mGy, representing only 0.9% of the LD_50/30_ (∼7 Gy) for BALB/c mice.^15^

A calibration scan of an acrylic cylinder with fiducials^16^ was performed before and after mouse scans. This process captures the tilt angle and center of rotation of the scan necessary for accurate CT reconstruction results. The source-to-isocenter of the rotation stage and source-to-detector distances were 374 and 3315 mm, respectively, resulting in an effective isotropic voxel size of 21.9 μm for the entire imaging system.

For 2D microangiography imaging, which was conducted after μCT imaging, an iodinated contrast agent (Isovue 370, Bracco Diagnostics, Princeton, NJ, USA; 370 mg iodine per ml) was injected via the jugular vein cannula with a microinjection pump (PHD-2000, Harvard Apparatus, Holliston, MA, USA) that was programmed to deliver a bolus administration of 0.12 ml of iodine contrast agents at a speed of 11 ml/min. Image acquisition was initiated 1 s before iodine injection, and 200 frames were recorded for each scan. The exposure time and frame rate of image acquisition were the same as that used for μCT imaging. The lung vessels were imaged when ventilation was interrupted for a 5-s breath hold at peak inspiration to eliminate any blurring from lung movement. Mice were given at least 5 min to recover from each injection of contrast agents. Angiography was performed three times for each mouse: right anterior oblique, left anterior oblique, and frontal views (without rotation during imaging), in increments of 45°.

### 2.C. 2D microangiography analysis

**FIG. 2.**
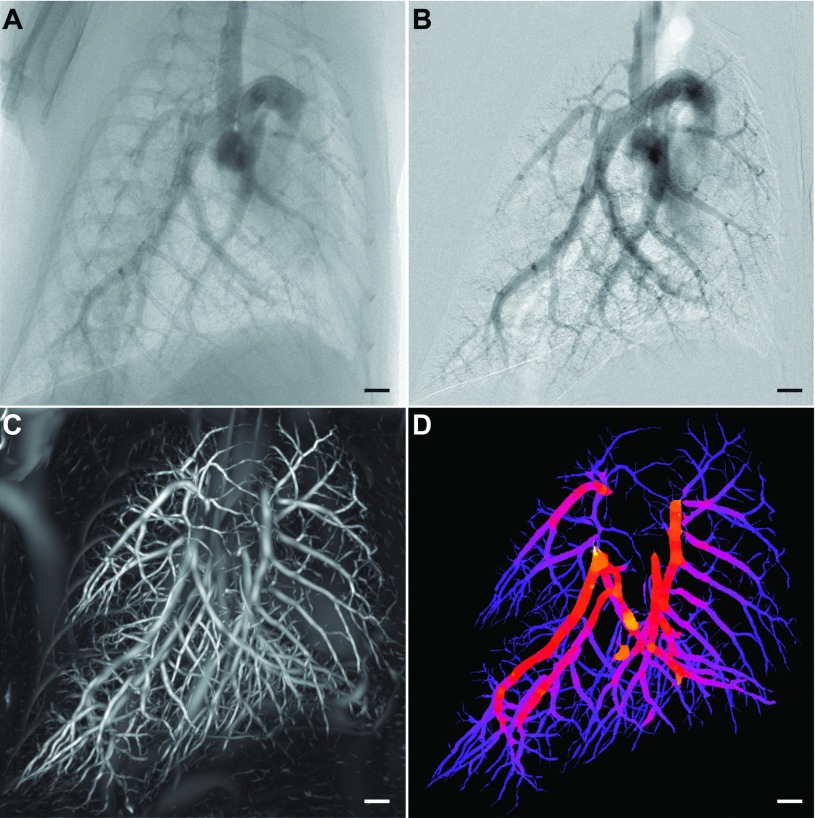
3D contrast-free diameter measurement of pulmonary vasculature. [(A) and (B)] 2D angiography with iodine bolus administration was used to validate our contrast-free vessel quantification method. (A) Unprocessed x-ray projection image with an iodinated contrast agent. Background correction and a moving average filter were used to enhance the contrast intensity in the image resulting in (B). (C) Maximum intensity projection through the probability volume (output from a Hessian-based vessel enhancement filter on a μCT volume without contrast agents) showing “vesselness.” (D) Maximum intensity projection of vasculature skeleton colored by our contrast-free vessel caliber measure. Scale bars represent 1 mm.

The sequence of 2D angiography images was analyzed using a combination of both custom in-house software and tools from fiji.^17^ Figure 2(A) shows an unprocessed image of the lungs after contrast injection acquired over a breath hold. A background correction using the averaged image of the entire sequence was performed to enhance the intensity of the iodinated vessels within the image. To stabilize the changes to pixel intensities from changes in relative iodine levels within the vessels, a temporal moving average filter (five images either side) was applied to the background corrected image sequence. A frame from the processed image sequence [Fig. 2(B)] in which the vessels were most intensely filled with contrast agents was chosen for quantitative 2D microangiography measurements. We used an automated plugin to fiji (Ref. 18) that measures the diameter of a vessel, *D*_ANG_, in an image using the full width at half-maximum approach. For a given line across a blood vessel, five measurements parallel to this line were obtained.

### 2.D. 3D CT image reconstruction and analysis

Image acquisition was synchronized with mechanical ventilation allowing image gating at peak inspiration when the contrast between lung tissue and adjacent blood vessels is maximal. A cone-beam reconstruction technique^19^ was used to obtain a single CT volume of the lungs at peak inspiration where the contrast between lung tissue and adjacent blood vessels is maximal [see Fig. 1(C) inset].

A vesselness image filter based on Frangi *et al.*^11^ was applied to this CT volume and the Hessian of the volume was calculated with a Gaussian kernel scale, *S*. Repeating over multiple scales, the local image gradients were matched to an ellipsoid to discriminate between plane-like structures and tubular structures. This filter produces a volume for the vesselness parameter, a measure relating to the likelihood that any given pixel belongs to a tubular structure, which we refer to as the probability volume. Figure 2(C) shows a maximum intensity projection of the 3D probability volume after applying the filter to a μCT reconstruction of mouse lungs. In the probability volume structures that are tubular have much higher contrast than other structures in the volume. This enables a more accurate segmentation of tubular structures within the volume in contrast to the unprocessed CT volume. A flood-fill segmentation using Avizo (FEI VSG, France) was used to segment the pulmonary vasculature from the computed probability volume and a skeletonization procedure^20^ was used to compute the centerline tree of the segmented pulmonary vasculature.

Our measures for vesselness were computed over a range of Gaussian kernel scales (1 ≤*S* ≤ 30). We also computed, for each voxel, the Gaussian scale (*S*_MAX_) that resulted in the highest value for vesselness which we captured in our probability volume. We refer to these data as the maximum kernel scale volume.

### 2.E. Calibration of Gaussian kernel scale to obtain vessel caliber

**FIG. 3.**
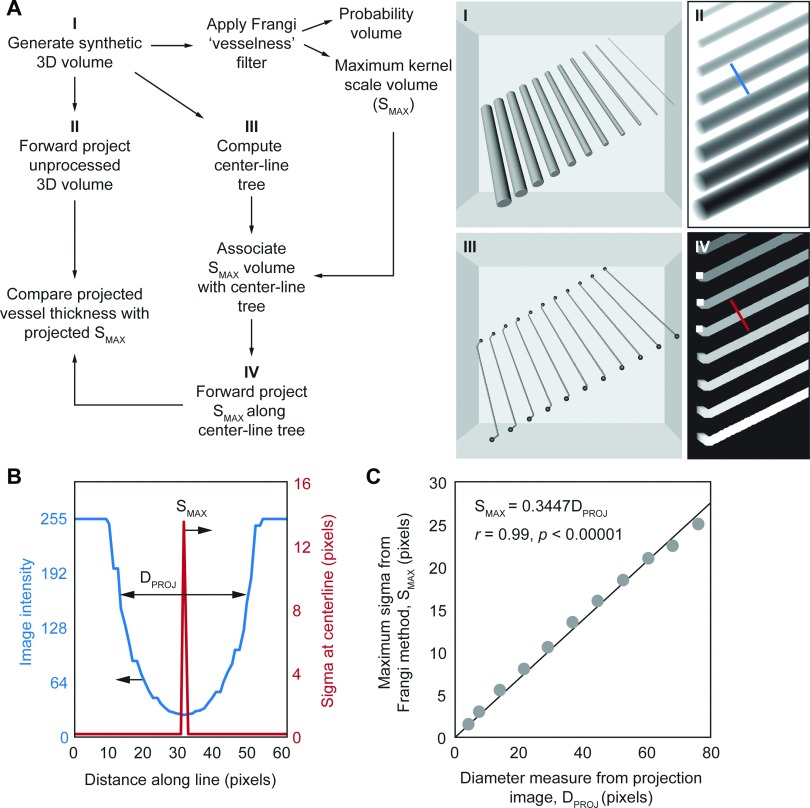
Calibration of contrast-free vessel caliber with automated intensity-based caliber measures. (A) Workflow for utilizing a synthetic volume of tubes to determine the correlation between *S*_MAX_, the maximum Gaussian kernel scale that maximizes the vesselness value in the Hessian-based vessel enhancement filter, and caliber measures from intensity projections of the volume. Forward projected centerline tree image in inset Sec. 4 has been thickened for clarity. (B) Example of the profiles across the line selection shown in insets Secs. 2 and 4 in (A). Vessel diameter in the intensity projected image (blue line), *D*_PROJ_, was measured using an automatic full width at half-maximum approach. *S*_MAX_ is the maximum value in the line selection across the tube in the *S*_MAX_ forward projection image (red line). (C) Plot of *S*_MAX_ plotted against *D*_PROJ_ measured across multiple sections along each of the tubes in the image.

Values for *S*_MAX_ were calibrated using computer-generated images of long tubes that varied in diameter (see Fig. 3). Tubes with diameters ranging between 3.14 and 62.8 px were placed in the volume and arbitrarily rotated in 3D (*x* = 30°, *y* = 15° and *z* = 0°) to ensure tubes are randomly aligned in the images. Figure 3(A) shows the flowchart of the processes used to filter the images and perform a quantitative comparison between the two techniques highlighted in Secs. 2.C and 2.D, in a similar manner to the experimental data captured using the laboratory x-ray imaging system.

The 3D volume was generated (Sec. 1) and a maximal intensity forward projection was performed to generate projection views of the tubes similar to our experimental 2D microangiography data. These projection images were analyzed using the automated fiji plugin (see Sec. 2.C) to obtain measures for tube diameter, *D*_PROJ_. The 3D volume was then processed using Frangi’s Hessian-based vessel enhancement filter to obtain the probability volume and the *S*_MAX_ volume. The centerline tree was generated using the probability volume (Sec. 3) and measures of *S*_MAX_ were mapped to the tree. These data were forward projected (Sec. 4) to match the intensity forward projected views of the synthetic volume obtained in Sec. 2.

Line selections were drawn across each tube to simultaneously obtain measures for *D*_PROJ_ off the intensity projections [Fig. 3(B) blue line] and *S*_MAX_ off the *S*_MAX_ projected image [Fig. 3(B) red line]. We found that *S*_MAX_ is an excellent surrogate for vessel caliber demonstrating a linear correlation (slope = 0.3447; *R* = 0.99) with automated vessel caliber measurements from intensity projection images [Fig. 3(C)].

### 2.F. Vessel diameter quantification and comparison

**FIG. 4.**
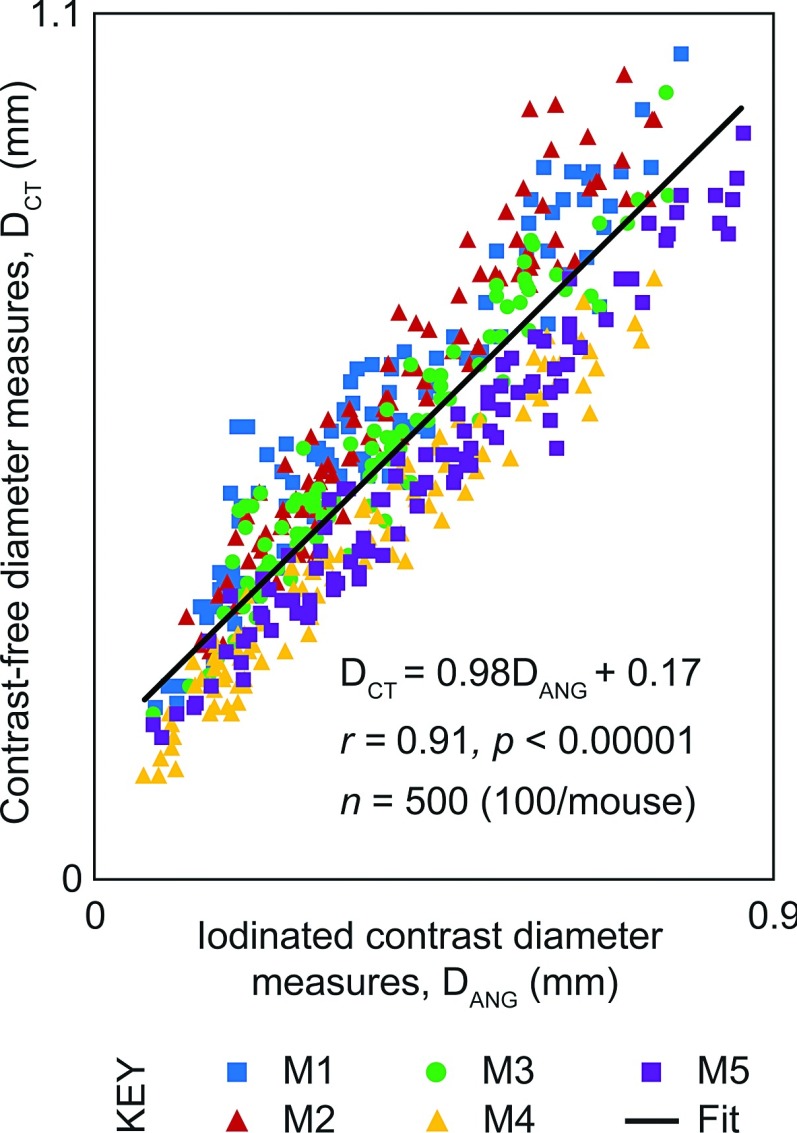
Quantitative contrast-free vessel caliber measures in experimental data. Vessel caliber measurements from contrast-free μCT in live mice, *D*_CT_, plotted against vessel diameter measured from 2D contrast microangiography images of the same mice.

The centerline tree of the segmented vasculature was utilized to map values of *S*_MAX_ to 3D points along the vascular tree. Values for *S*_MAX_ were converted to contrast free vessel caliber estimates, *D*_CT_, using the calibration results discussed in Sec. 2.E. This 3D volume was forward projected in increments of 45° to match the right anterior oblique, left anterior oblique, and frontal view obtained with 2D contrast microangiography. A typical resulting composite image of the segmented vasculature tree colored by *D*_CT_ is shown in Fig. 2(D). Line selections were drawn across blood vessels at multiple points in the image and measures *D*_CT_ and *D*_ANG_ (see Sec. 2.C) were obtained. Figure 4 shows that our contrast-free measures for vessel caliber, *D*_CT_, show good correlation with quantitative 2D microangiography measures for vessel caliber, *D*_ANG_ (slope = 0.98; intercept = 0.17 mm; *R* = 0.91).

### 2.G. Statistical methods

Linear regression was used to determine the relationship between *S*_MAX_ and *D*_PROJ_ using synthetic data [Fig. 3(C)]. Each point on Fig. 3(C)] represents an average of 25 measurements. This relationship was used to calibrate measures for *S*_MAX_ to determine contrast-free vessel caliber, *D*_CT_, obtained from *μ*CT images of individual mice. Linear regression was used to determine the relationship between *D*_CT_ and caliber measures from 2D contrast microangiography, *D*_ANG_ (Fig. 4) using *n* = 500 measures (*n* = 100/mouse).

## 3. RESULTS AND DISCUSSION

BALB/c mice (*n* = 5) were imaged using a laboratory x-ray source (see methods) under mechanical ventilation [Figs. 1(A) and 1(B)] using a small-animal ventilator (see methods). Gated-μCT imaging of breathing mice [Fig. 1(C)] was performed resulting in a single CT reconstruction of the murine lungs at peak inspiration. Hessian-based enhancement filters^11^ are gaining popularity for vessel segmentation.^8,10,21^ Most studies focusing on quantifying the pulmonary vasculature are based on humans.^9^ To the best of our knowledge, no other group has quantified murine pulmonary vessel caliber using CT images without contrast agents.

Here, we apply a multi-scale Hessian-based enhancement filter to obtain vessel caliber measurements from CT images [Fig. 2(D)]. Figure 2(E) shows the composite image that incorporates the Gaussian scale used for vessel enhancement and the segmented pulmonary vasculature. We first calibrated the Gaussian kernel scale parameter using synthetic images of tubes with known diameters [Figs. 3(A) and 3(B)]. Using a similar analysis protocol to the experimental data, the Gaussian kernel scale measure was calibrated against vessel diameter measured from 2D intensity projection images of the synthetic tubes [Fig. 3(C)].

Our method was validated using 2D microangiography with contrast agents [Figs. 2(A) and 2(B)] in the same mice from three different views. The images are pre-processed using a digital subtraction^22^ and averaging scheme to enhance the intensity of the pulmonary arteries. Figures 2(A) and 2(B) shows images obtained from microangiography before and after processing, respectively. Figures 2(A) and 2(B) are approximately ∼0.6 s after contrast administration. Failure to correctly place the catheter tip at the superior vena cava is one disadvantage of the conventional contrast microangiography approach that is eliminated with our new CT approach.

We probed corresponding images to obtain measures for vessel diameter, *D*_CT_ (after applying the calibration from synthetic testing), and vessel diameter from 2D angiography images (*D*_ANG_) at varying locations along the pulmonary vasculature (Fig. 4). Both pulmonary arteries and veins were segmented from the filtered CT volumes as evident in the image. Up to 16 generations of branches within the vasculature tree were visible in the segmented vasculature. With 2D angiography, a contrast medium was only highly visible in the arteries following a bolus injection. As a result, only pulmonary artery caliber measures were validated in this study. A total of 500 pulmonary arterial diameter measurements were obtained for five mice with a fixed number of 100 measurements per mouse. Our results show that there is a good correlation between the vessel caliber measurements from 2D contrast angiography and our contrast-free CT method (*R* = 0.91). The scatter of the points on the plot suggests that there are inaccuracies in both techniques used in this study. However, more importantly much of this scatter and the associated non-zero intercept may be the cause of a more fundamental difference in the two techniques. There is no significant difference between the x-ray absorption of blood and the arterial wall. As a result, the Hessian-based vessel enhancement approach used here increases the contrast in tubular structures between lung tissue (predominantly air) and other structures that are predominantly water [see Fig. 1(c) inset]. Thus, this would suggest that our approach detects the outer diameter of blood vessels, while the 2D contrast microangiography approach detects the inner diameter of blood vessels where there is an iodine-enhanced contrast. More work is warranted to assess the clinical applications of this approach which is outside of the scope of this study. Importantly, an alternative approach may be to utilize both CT pulmonary angiography (CTPA) and CT to gauge wall thickness and vascular remodeling as well as overall vessel morphology.

## 4. CONCLUSIONS

A tool for obtaining vessel caliber measures from (contrast free) CT images is presented. The laboratory-based imaging system used here yields images with sufficient resolution to resolve blood vessels *in vivo* with diameters ranging from 62 μm and above in mice. The imaging system and the tools discussed here present an alternative angiography technique for small animal studies without the need for contrast agents, and theoretically could allow repeat imaging in the same animals over time as disease processes progress or in response to treatment.

Although this technique was pursued to permit better analysis of pulmonary perfusion patterns in murine models of lung disease, the obvious clinical applications cannot be overlooked. Acute pulmonary embolism (PE) is the migration of a blood clot from a deep vein in an extremity (usually the legs) through the right side of the heart and into the pulmonary arteries. PE is a common and sometimes fatal condition with mortality rates ranging from 14% to 28%,^23^ to achieve the best outcomes for patients, PEs require rapid diagnosis and treatment.^24–27^ Clinical signs and symptoms provide some clues towards a diagnosis of PE but ultimately a definitive diagnosis of PE requires imaging. Contrast-enhanced CT arteriography has rapidly replaced ventilation-perfusion scanning as the imaging modality of choice for diagnosing PE, especially with the advent of multi-detector CT scanners that allow analysis of the pulmonary arteries and detection of emboli at the subsegmental level.^26,28–30^ However, CT angiography can cause contrast medium-induced nephropathy (CIN), which is the third most common cause of in-hospital acute renal failure,^31–33^ conferring significant morbidity and mortality. Therefore, a method of imaging the pulmonary vasculature without the use of contrast would represent a dramatic improvement in the risk/benefit profile of diagnostic testing for all patients suspected of having PEs and especially those who are at risk for CIN.

For the first time, we demonstrate a non-contrast approach to generate high fidelity, 3D quantification of the pulmonary vasculature in a murine model. It is not inconceivable that such a technique could be applied to human subjects for clinical applications including the detection of abnormalities in the pulmonary vasculature that correlate with PE. It is important to note that the radiation dose received by mice in this study is not high (∼60 mGy), especially when considered relative to the LD_50/30_ (∼7 Gy). Additionally, the scale of the vasculature in the mouse lung necessitates the use of a specialized microfocus x-ray source (such as liquid-metal-jet anode used here), to obtain the required resolution. A consequence of a liquid-metal anode is an energy spectrum that is weighted with low energy x-rays, contributing to a higher measured absorbed dose. When scaling the subject from the size of a mouse to a human, the concomitant scaling of the imaging technology allows features of the same relative size to be imaged with lower dose. We are confident that the resolution currently used in CTPA systems is sufficient for the clinical application of this technique, with a dose less than 7 mSv.^34^ Studies to this end are currently underway.
